# Cancer Accumulation and Anticancer Activity of “CROX (Cluster Regulation of RUNX)” PIP in *HER2* ‐Positive Gastric Cancer Evaluated by Chicken Egg Cancer Model

**DOI:** 10.1002/cam4.70845

**Published:** 2025-04-02

**Authors:** Tatsuya Masuda, Takayoshi Watanabe, Yasutoshi Tatsumi, Jason Lin, Kazuhiro Okumura, Toshinori Ozaki, Hiroshi Sugiyama, Yasuhiko Kamikubo

**Affiliations:** ^1^ Division of Molecular Carcinogenesis Chiba Cancer Center Research Institute Chiba Japan; ^2^ Department of Human Health Sciences, Graduate School of Medicine Kyoto University Kyoto Japan; ^3^ Division of Cancer Genetics Chiba Cancer Center Research Institute Chiba Japan; ^4^ Division of Experimental Animal Research Cancer Genome Center, Chiba Cancer Center Research Institute Chiba Japan; ^5^ Institute for Integrated Cell‐Material Sciences, Institute for Advanced Study Kyoto University Kyoto Japan

**Keywords:** CAM assay, cancer cell accumulation, drug delivery, nuclear localization, pyrrole‐imidazole polyamide, RUNX inhibitor

## Abstract

**Background:**

We have focused on pyrrole‐imidazole (PI) polyamide compounds, which preferentially bind to their target DNA sequences. To validate our “CROX (Cluster Regulation of RUNX)” strategy, we have created a novel PI polyamide‐based inhibitor against RUNX termed Chb‐M’. Recently, we have confirmed its cancer‐specific uptake in mouse xenograft derived from *HER2*‐positive gastric cancer cells. The accumulation and efficacy of Chb‐M' in cancer has not yet been investigated *in vivo*, which is a simpler and less expensive method other than mouse xenograft models.

**Methods:**

In the present study, we have employed the simple and versatile experimental system termed CAM (chorioallantoic membrane) model, and evaluated whether Chb‐M’ could have the cancer accumulation potential and anti‐cancer activity.

**Results:**

Based on our present results, gastric cancer MKN45 cells transplanted onto CAM successfully developed cancers, and the intravenously injected FITC‐labeled Chb‐M’ obviously accumulated in these CAM cancers. As expected, the treatment of the CAM cancers with Chb‐M’ significantly attenuated the growth of the CAM cancers. Our present results were basically identical to those obtained from mouse xenograft model.

**Conclusion:**

Our present findings strongly suggest that Chb‐M’ preferentially accumulates in cancer to suppress its growth, and the CAM model might serve as a valuable and promising platform to rapidly assess the cancer uptake and anti‐cancer efficacy of various PI polyamide‐based drug candidates.

## Graphical abstract

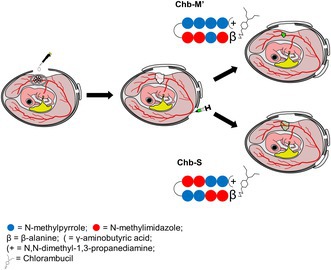

## 1 Introduction

From the clinical point of view, targeted delivery of the candidate anticancer drugs into cancers is quite important for cancer therapy. The CAM (chorioallantoic membrane) model has been considered to be a simple system for the evaluation of the drug accumulation within cancers. The CAM model employs the fertilized chicken eggs. Ten days after incubation, the window is opened in the eggshell and the cancer cells are transplanted onto the CAM. Due to the nutrient‐rich and highly angiogenic nature of the CAM of the fertilized chicken eggs, the transplanted cancer cells rapidly form the histochemically identifiable cancers in a few days. The advantages of the CAM model are as follows: First, the chicken embryo is immunodeficient at the early stages and can accept cancer cells regardless of their origin without an immune response. Cancers such as glioblastoma and renal cell carcinoma display a higher uptake rate from 80% to 100%, and the changes in morphology of cancer cells are easily observed by microscopy. Second, despite a short observation period, the CAM cancers histologically resemble primary cancers and exhibit morphological characteristics of the original cancers. Third, the cost of the equipment and the eggs themselves is quite low. Fourth, the rapid development of cancers. It takes only a few days to observe cancers in the CAM, while it takes several weeks in the mouse. On the other hand, the major disadvantages of the CAM model are as follows: First, the posttreatment observation period is short. The window for CAM experimentation lasts only 10 days. Second, there are differences in terms of the drug's metabolism and the immune system between chickens and mammals [1, 2, 3, 4, 5, 6, 7].

Runt‐related transcription factor 1 (RUNX1), also known as acute myeloid leukemia 1 protein (AML1), is a member of the core‐binding factor family of transcription factors (RUNX family) and indispensable for the establishment of the definitive hematopoiesis [8]. The recent studies strongly suggest that wild‐type RUNX1 is required for the proliferation and survival of certain types of leukemia cells [9]. The presence of the compensation mechanism among RUNX family members (RUNX1, RUNX2, and RUNX3) [10, 11] prompted us to imagine that targeting whole RUNX family could be an effective strategy to suppress the malignant phenotypes of leukemia cells. We referred this strategy as to “CROX (cluster regulation of RUNX)” [10, 12].

To evaluate our CROX strategy, we have created Chb‐M' [10, 13] based on pyrrole‐imidazole (PI) polyamide, which binds to the target sequences of the transcription factors and efficiently attenuates their transactivation ability [14, 15, 16]. Chb‐M' is a PI polyamide interlocked with a hairpin conjugated with the alkylating reagent chlorambucil that preferentially targets the consensus RUNX‐binding sequence, 5′‐TGTGGT‐3′. Since Chb‐M' preferentially inhibited the binding of RUNX family members to their consensus‐binding sequence, it is likely that Chb‐M' might efficiently switch off RUNX‐target pro‐oncogenic genes [10, 11, 12, 13, 17, 18, 19, 20, 21, 22, 23, 24, 25]. As expected, our previous studies demonstrated that Chb‐M' has a remarkable anticancer effect on a variety of cancers including AML, Ph^+^ ALL, lung cancer, and MRT in vitro and in vivo [10, 18, 19, 20, 21]. In addition, we reported that RUNX1 regulates ErbB2/HER2 signaling pathway in *HER2*‐positive gastric cancer cells through transactivating SOS1 expression, rendering itself an ideal target for antitumor strategy toward this cancer. Thus, we have identified a novel interaction of RUNX1 and the ErbB2/HER2 signaling pathway. According to our observations, Chb‐M' was a novel drug candidate that enables the CROX approach, consistently inactivating the ErbB2/HER2 signaling pathway, and is effective on several *HER2*‐positive gastric cancer cell lines [17]. Based on our previous results obtained from mouse xenograft derived from *HER2*‐positive gastric cancer cells, we have found the cancer‐specific uptake of Chb‐M' [10]. Therefore, we have sought to employ the CAM model, and then establish a *HER2*‐positive gastric cancer cell transplantation model in a short period of time, which could accelerate the validation of the anticancer effect and its accumulation in cancer tissues. If we could obtain a favorable result, our present study strongly suggests that the CAM model serves as a valuable and promising platform to contribute to the screening of various PI polyamide‐based drug candidates.

## 2 Materials and Methods

### 2.1 Cell Lines

Human gastric cancer‐derived MKN45 and NUGC4 cells were used in the present study. MKN45 cells were kindly gifted by Dr. M. Muto (Kyoto University, Japan). NUGC4 cells were purchased from the Japanese Collection of Research Bioresources (JCRB) Cell Bank, Japan. These cells were cultured in RPMI 1640 medium (Nacalai Tesque, Kyoto, Japan) supplemented with heat‐inactivated 10% fetal bovine serum (Serana, Brandenburg, Germany) and 1% of penicillin–streptomycin (Nacalai Tesque) at 37°C in a humidified atmosphere of 5% CO_2_.

### 2.2 Synthesis of FITC‐Conjugated Chb‐S and Chb‐M'

Reagents and solvents were purchased from the standard suppliers and used without further purification. Automated polyamide synthesis was performed on the PSSM‐8 system (Shimadzu) as described previously [26]. HPLC analysis of each synthesized compound was performed on the Jasco Engineering PU‐2089 plus series system using the Chemcobond 5‐ODS‐H 4.6 mm × 150 mm column (Chemco Plus Scientific) in 0.1% TFA in water with acetonitrile as the eluent at a flow rate of 1.0 mL/min and a linear gradient elution of 0%–100% acetonitrile in 40 min with detection at 254 nm. The collected fractions were analyzed by MALDI‐TOF‐MS Microflex‐KS II (Bruker).

The building blocks used in this study were FmocHN‐Py‐CO_2_H, FmocHN‐Im‐CO_2_H, FmocHN‐PyIm‐CO_2_H, BocHN‐β‐alanine‐CO_2_H, and FmocHN‐γ‐aminobutyric acid. Each of them was introduced sequentially to 106 mg of Py‐oxime resin (0.463 mmol/g). Following the cleavage reaction with 1 mL of 3,3′‐diamino‐*N*‐methyldipropylamine at 55°C for 3 h, filtration and Et_2_O precipitation gave 80.7 mg of the crude polyamide. The crude sample was dissolved in 400 mL of DMF and purified by Flash column purification (TELLEDYNE ISCO) to obtain 61.3 mg of the Boc‐protected precursor. 11.4 mg of the Boc‐protected precursor was dissolved in 342 μL of DMF, added FITC (6.5 mg, 17 μmol) plus DIEA (6.5 mg, 50 μmol), and then the reaction mixture was stirred at room temperature for 2 h. Et_2_O precipitation gave the crude FITC‐polyamide (12.6 mg). The FITC‐polyamide was treated with 1 mL of 4 N HCl/EtOAc at room temperature for 2 h. The resulting product was treated with chlorambucil (5.1 mg, 17 μmol), PyBOP (8.7 mg, 17 μmol), and DIEA (6.5 mg, 50 μmol) in 342 μL DMF at room temperature for 2 h. After Et_2_O precipitation, the crude sample of FITC‐Chb‐M' was obtained. This crude sample was dissolved in DMF and purified by HPLC to finally obtain pure FITC‐Chb‐M' as a white powder (4.4 mg). An analogous procedure as FITC‐Chb‐M' gave FITC‐Chb‐S, FITC‐Chb‐S as a white powder (3.5 mg).

### 2.3 Nuclear Targeting of FITC‐Chb‐M'

MKN45 and NUGC4 cells were seeded at a density of 3 × 10^5^ cells/mL into 8‐well chamber mounted on a glass slide (ibidi, Gräfelfing, Germany). Twenty‐four hours postincubation, cells were treated with 1 μM of FITC‐Chb‐M', FITC‐Chb‐S, or with FITC (Dojindo Laboratories, Kumamoto, Japan). DMSO was used as a negative control. Two hours after treatment, cells were washed twice in ice‐cold PBS and fixed with 4% paraformaldehyde for 10 min at room temperature. After fixation, coverslips were mounted with VECTASHIELD Antifade Mounting Medium containing DAPI (Vector Laboratories, California, USA). Fluorescence images were observed under a Leica TCS SP8 confocal laser microscope (Leica Microsystems, Wetzlar, Germany).

### 2.4 Cancer Accumulation and Nuclear Access of FITC‐Chb‐M'

MKN45 and NUGC4 cells were seeded at a density of 6 × 10^5^ cells/well on a 6‐well plate. Twenty‐four hours after incubation, 1 μM of FITC‐Chb‐M', FITC‐Chb‐S, or FITC was applied to these cells. DMSO was used as a negative control. Following 2 h of treatment, cells were washed twice in PBS, and whole cell lysates or nuclear lysates were prepared by using lysis buffer (Cell Signaling Technology, Massachusetts, USA) or Nuclear Protein Isolation Kit (Enzo Life Sciences, New York, USA), respectively. Their fluorescence intensity was determined by ARVO X3 multimode plate reader (PerkinElmer, Massachusetts, USA). The wavelength of excitation and fluorescence emission of FITC was 485 and 535 nm, respectively.

### 2.5 Establishment of the CAM Model

The fertilized white chicken eggs (purchased from Japan Layer, Gifu, Japan) were incubated in a bird incubator (AUTOELEX, Gyeongsangnam‐do, Korea) at 37.5°C and 65% humidity with one turn per hour. Ten days after incubation, the indicated cancer cells were transplanted onto them. Firstly, the blunt end of the eggshell (above the air cell) was deeply cut with a diamond cutter. Subsequently, the window was opened in the eggshell without any damage to the shell membrane. The shell membrane was removed, and the CAM was dropped. To transplant the cancer cells, we used a sterile Teflon ring (Tokyo Garasu Kikai, Tokyo, Japan) that was placed at the Y‐shaped blood vessel on the CAM. The cancer cells (2 × 10^6^ cells/20 μL medium/egg) were added to the ring, and then the window was covered with Tegaderm film (3M Japan, Tokyo, Japan). After returning the eggs to the incubator, the egg rotation setting was turned off. On Day 12, the Teflon ring was removed from the CAM. To confirm the engraftment, MKN45 cells transduced with a lentiviral vector (CS‐RfA‐ETV, RIKEN BioResource Center) that constantly express Venus fluorescent protein were transplanted onto the CAM. The CAM model experiments did not require any special allowances as long as the embryos were sacrificed before hatching.

### 2.6 Cancer Accumulation of FITC‐Chb‐M' in the CAM Model

The small window was opened in the chicken egg on Day 14 without damaging the shell membrane. FITC‐Chb‐M' (32 μg/egg), FITC‐Chb‐S (32 μg/egg), FITC (equivalent molar amount), or DMSO (equivalent volume) was then injected intravenously. On Days 14 and 15 (before injection, 3, 6, 12, and 24 h after injection), the window of the eggs, resected tumors, and each organ of the embryo (heart, liver, spleen, lung, kidney, brain, intestine, and stomach) were photographed under a Leica MZ16FA fluorescence stereomicroscope and K5C digital camera (Leica Microsystems) to examine the organ distribution of each drug. ChemiDoc Touch MP (Bio‐Rad Laboratories, California, USA) was used to quantify the fluorescence intensity in each organ with a fluorescein filter. The wavelength of the excitation and fluorescence emission of fluorescein was 460–490 and 518–546 nm, respectively. In addition, the representative cancers developed on the CAM were fixed overnight in 4% paraformaldehyde at 4°C, treated with 99.8% methanol for 30 min at −80°C, incubated in a 20% sucrose solution overnight at 4°C, embedded in OCT compound at −80°C, thinly sliced by a cryomicrotome to approximately 10 μm in thickness, washed in ice‐cold PBS, mounted with mounting medium containing DAPI, and then observed under a Leica TCS SP8 confocal laser microscope.

### 2.7 Treatment of the Cancers Developed on the CAM With Chb‐M'

The small window was opened in the chicken egg on Day 13 without damaging the shell membrane. Chb‐M' (6.4 μg/egg), Chb‐S (6.4 μg/egg), or DMSO (equivalent volume) was then injected intravenously. On Day 16, the eggs were photographed under a Leica S9i stereomicroscope (Leica Microsystems) to examine the anticancer effects of each drug. The CAM cancers were resected, weighed, and fixed overnight in 4% paraformaldehyde. Cancers were photographed to compare each experimental group by using the Leica S9i stereomicroscope. Paraffin‐embedded blocks were prepared using a paraffin‐embedding device CT‐Pro20 (Genostaff, Tokyo, Japan).

### 2.8 Immunohistochemical Staining

Paraffin‐embedded blocks were sliced to approximately 6 μm in thickness. After deparaffinization, the slice was heated in a microwave and incubated with the primary antibodies, such as rabbit monoclonal antibody against human SOS1 (abcam, AB140621) and normal rabbit IgG (Dako, X0936). After incubation, antirabbit IgG Biotin (Vector, BA‐1000) was added, and color was developed with PO‐labeled streptavidin and DAB/hydrogen peroxide. Pathological images were observed using a virtual slide scanner NanoZoomer (Hamamatsu Photonics, Shizuoka, Japan).

### 2.9 TUNEL Staining

Paraffin‐embedded blocks were sliced to approximately 6 μm in thickness. After deparaffinization, TUNEL staining was performed using In situ Apoptosis Detection Kit (Takara, MK500) according to the manufacturer's instructions. After the reaction, DAB/hydrogen peroxide was added to develop the color. Pathological images were observed using a virtual slide scanner NanoZoomer.

### 2.10 Calculation of Number of the Putative‐Binding Sites of PI Polyamide Using hg19 Human Reference Genome

FASTA sequences for the hg19 human reference genome were obtained per chromosome from the Human Genome Sequence and annotation download archives available from the University of California Santa Cruz Genomics Institute; only complete contigs were used for the purpose of motif determination. Motifs corresponding to Chb‐S (5′‐WGGCCW‐3′) and Chb‐M' (5′‐WGWGGW‐3′ and its complement motif 5′‐WCCWCW‐3′) were then searched against the hg19 reference genome using a custom Perl (version 5.16) script developed as part of the rfPIPeak package (available at https://github.com/jlincbio/rfpipeak); results were then compiled into BED format for counting and reporting purposes.

### 2.11 Statistical Analysis

All in vitro data and the data obtained from the chicken egg experiments were statistically analyzed by the two‐tailed Student's *t*‐test and expressed as mean ± SEM values of independent experiments. *p*‐values < 0.05 were considered to be significant.

### 2.12 Study Approval

All CAM model studies were properly conducted in accordance with the Regulation on Animal Experimentation at Chiba Cancer Center Research Institute, based on International Guiding Principles for Biomedical Research Involving Animals. All procedures in this study were approved by Kyoto University Animal Experimentation Committee (permit number: 24‐1).

## 3 Results

### 3.1 Synthesis of FITC‐Conjugated Chb‐S

**FIGURE 1:**
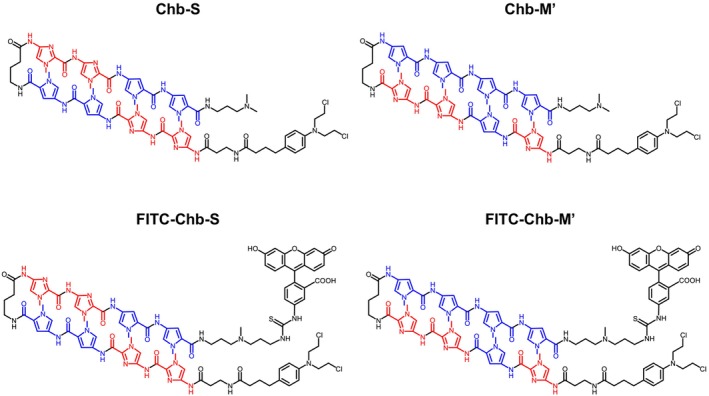
Chemical structure of Chb‐S, Chb‐M', FITC‐Chb‐S, and FITC‐Chb‐M'. Chb‐M' is a RUNX consensus sequence‐targeting DNA‐alkylating drug, and Chb‐S is a mismatch drug, which has no binding motif within RUNX consensus sequence. FITC‐Chb‐S and FITC‐Chb‐M' are fluorescently labeled with FITC at the C‐terminus of Chb‐S and Chb‐M', respectively. *N*‐methylpyrrole and *N*‐methylimidazole are indicated in blue and red, respectively.

As described previously [10, 17, 19, 20, 21, 22, 23, 24], we have produced Chb‐S (PI polyamide which targets 5′‐WGGCCW‐3′ sequence and randomly binds to DNA [10]). In the present study, we have employed this Chb‐S as a negative control for Chb‐M'. Although we have already generated FITC‐conjugated Chb‐M' (FITC‐Chb‐M') and confirmed its incorporation into cancer cells in vitro and in vivo [10], Chb‐S has not yet been examined so far. To check their cancer accumulation, organ distribution, and anticancer efficacy in the CAM model, we have newly synthesized FITC‐Chb‐S as shown in Figure 1 and Figure S1.

### 3.2 Nuclear Access of Chb‐M'

**FIGURE 2:**
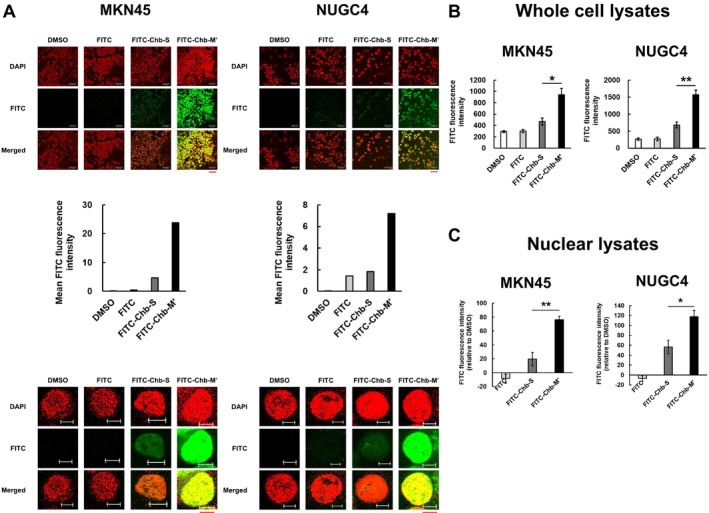
Nuclear access of FITC‐Chb‐M'. (A) Nuclear localization of Chb‐M'. MKN45 (left) and NUGC4 (right) cells were treated with 1 μM of FITC‐Chb‐M', FITC‐Chb‐S, or with FITC. DMSO was used as a negative control. Two hours after treatment, cell nucleus was stained with DAPI (red). Fluorescence images (green) were captured under the confocal laser microscope (magnitude: ×400). Merged images were shown in yellow. Scale bars indicate 50 μm. Bar graphs show mean FITC fluorescence intensity. Higher magnification of images was shown in the bottom panels. Only one cell was displayed. Scale bars indicate 5 μm. (B) Quantitation of the signal intensity. MKN45 and NUGC4 cells were treated as in (A). Two hours after treatment, whole cell lysates were prepared and their fluorescence intensities were determined by the fluorescence plate reader (*n* = 3). Data are the mean ± SEM values. A significant difference test was performed between the signal intensities arising from FITC‐Chb‐S and FITC‐Chb‐M' in MKN45 (left) and NUGC4 (right) cells. *p < 0.05, **p < 0.01 (two‐tailed Student's *t*‐test). (C) MKN45 and NUGC4 cells were treated as in (A). Two hours after treatment, nuclear lysates were prepared and their fluorescence intensities were determined by the fluorescence plate reader (*n* = 3). Data are the mean ± SEM values. Each value is normalized with DMSO. *p < 0.05, **p < 0.01 (two‐tailed Student's *t*‐test).

Firstly, we have checked the subcellular localization of Chb‐M'. To this end, FITC‐Chb‐M', FITC‐Chb‐S, FITC alone, or DMSO was added to the culture of human gastric cancer MKN45 and NUGC4 cells. FITC‐Chb‐S was used as an appropriate negative control. Two hours after treatment, cells were observed under confocal microscopy. As clearly seen in Figure 2A, FITC‐Chb‐M' but not FITC‐Chb‐S was localized in cell nucleus of MKN45 and NUGC4 cells. Consistent with these observations, the quantification of the fluorescent signals emitted from FITC revealed that a large amount of FITC‐Chb‐M' is detectable in whole cell lysates and nuclear lysates prepared 2 h after treatment (Figure 2B,C). These results strongly suggest that Chb‐M' but not Chb‐S significantly accumulates within the gastric cancer cell nucleus.

### 3.3 Intra‐Cancer Uptake of FITC‐Chb‐M' in Fertilized Chicken Eggs

**FIGURE 3:**
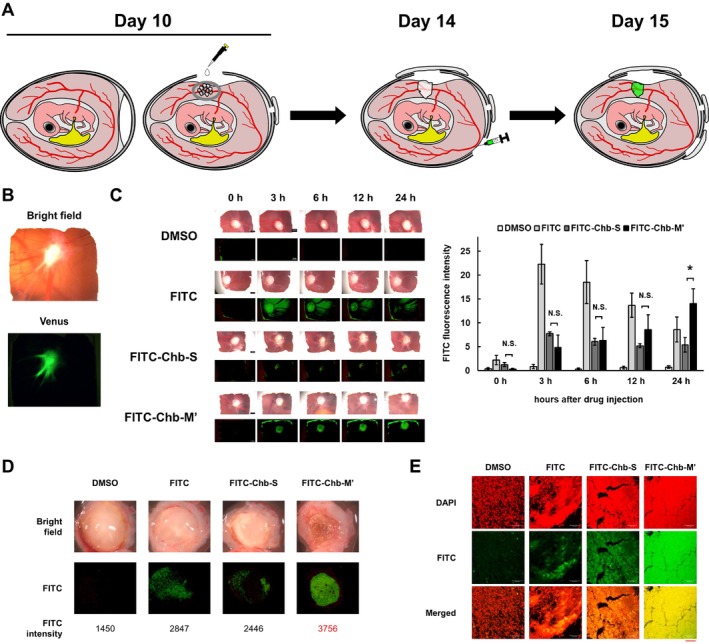
Intra‐cancer uptake of FITC‐Chb‐M' in fertilized chicken eggs transplanted with MKN45 cells. (A) A graphical drawing of the chicken egg cancer model. To examine the cancer accumulation of Chb‐M' and Chb‐S, FITC‐labeled Chb‐M' or FITC‐labeled Chb‐S was intravenously injected into the chicken eggs (Day 14). Before injection, 3, 6, 12, and 24 h after injection, the window of the eggs, resected cancers, and each organ of the embryo were observed under the stereoscopic fluorescence microscope. (B) Fluorescence image (taken with GFP filter) of Venus‐expressing MKN45 cells transplanted onto CAM (Day 13). (C) Representative bright‐field and FITC fluorescence (taken with GFP filter) images of the egg windows obtained from the indicated control and experimental groups (before injection, 3, 6, 12, and 24 h after injection). Right bar graphs show time course of the FITC fluorescence intensity of cancers on the CAM in each experimental group (*n* = 5). Data are the mean ± SEM values. A significant difference test was performed between the signal intensities arising from FITC‐Chb‐S and FITC‐Chb‐M'. *p < 0.05; N.S., no significant (two‐tailed Student's *t*‐test). (D) Representative bright‐field and FITC fluorescence (taken with GFP filter) images of the resected cancers obtained from the indicated control and experimental groups (24 h after injection). The values below indicate the fluorescence intensity of FITC. (E) DAPI staining and FITC fluorescence images of frozen sections of MKN45 cancers resected from the CAM. Merged images (yellow) are shown in the bottom panels. Scale bars indicate 50 μm.

We then sought to ask whether Chb‐M' could preferentially accumulate within cancers in vivo. As described previously, FITC‐Chb‐M' obviously accumulated in cancers arising from MKN45 cells transplanted into BALB/c‐nu mice [10]. In this study, we have employed an alternative and simple experimental system based on the fertilized chicken eggs (CAM model) to assess its cancer accumulation potential and anticancer activity. The experimental design was shown in Figure 3A. Firstly, MKN45 cells transduced with a lentiviral vector encoding Venus fluorescent protein (the derivative of GFP) were transplanted onto the CAM and their engraftment was confirmed (Figure 3B). After that, the eggshell was windowed and MKN45 cells were transplanted onto the CAM (Day 10). On Day 14, FITC‐Chb‐M' (32 μg/egg), FITC‐Chb‐S (32 μg/egg), FITC alone (equivalent molar amount), or DMSO (solvent) was intravenously injected into the chicken eggs. At the indicated time periods after injection, the window of the eggs was observed with the stereoscopic fluorescence microscope, and the CAM cancers as well as each organ of the embryo were resected 24 h after injection. As shown in Figure 3C and Figure S2, the intensity of the signal emitted from FITC‐Chb‐M' and FITC alone increased and decreased in a time‐dependent manner, respectively. In contrast, the intensity of the signal arising from FITC‐Chb‐S remained basically unchanged after injection. The close inspection of the resected cancers treated with the indicated compounds revealed that the FITC signal emitted from the CAM cancers exposed to FITC‐Chb‐M' is stronger than those treated with DMSO, FITC alone, or with FITC‐Chb‐S (Figure 3D). Among the frozen sections prepared from the indicated CAM cancers, FITC‐Chb‐M'‐treated CAM cancers showed the strongest FITC signal and the merged image indicated its nuclear access (Figure 3E). Thus, it is likely that Chb‐M' but not Chb‐S largely accumulates in the cancer cell nuclei.

### 3.4 Preferential Accumulation of FITC‐Chb‐M' in the CAM Cancers but Not in Normal Organs

**FIGURE 4:**
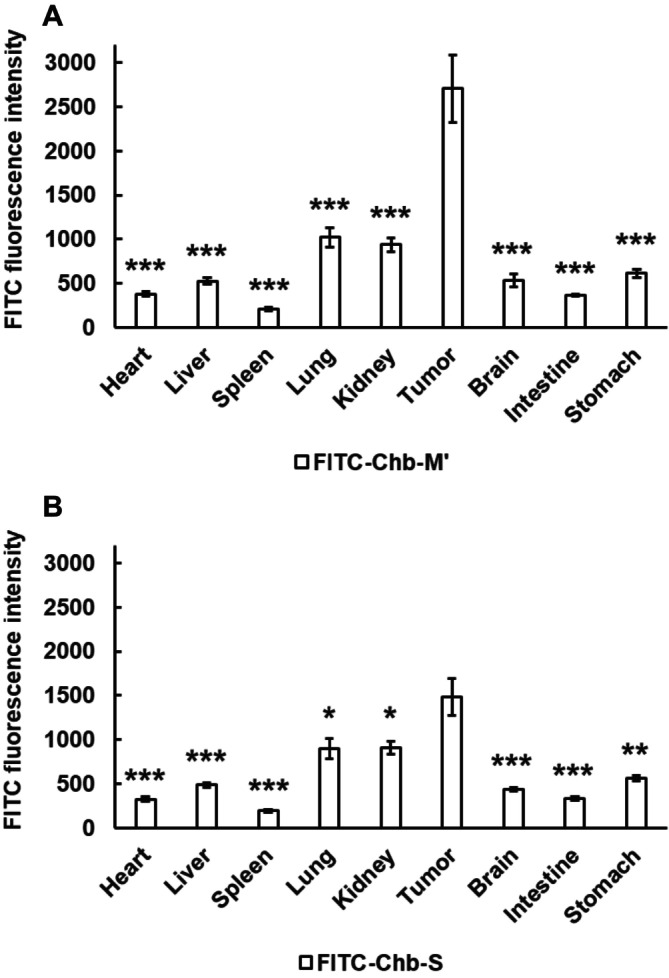
Preferential accumulation of FITC‐Chb‐M' in the CAM cancer. FITC fluorescence intensity emitted from each organ and the CAM cancers in response to FITC‐Chb‐M' (A) or FITC‐Chb‐S (B) was quantitated (*n* = 11). Data are the mean ± SEM values. *p* value was obtained by two‐tailed Student's *t*‐test. *p < 0.05, **p < 0.01, ***p < 0.001.

To further confirm the results showing the preferential accumulation of Chb‐M' in cancers, we have examined the distribution of the indicated drugs in the organs resected from the CAM models. As clearly seen in Figure 4A,B, ChemiDoc Touch MP‐mediated quantification of FITC fluorescence intensity revealed that the signal arising from FITC‐Chb‐M' obviously accumulates within the CAM cancers relative to FITC‐Chb‐M'‐treated organs, whereas FITC‐Chb‐S is detectable in cancers but to a lesser degree. Consistent with these observations, the signal emitted from FITC‐Chb‐M' was largely restricted to the CAM cancers as compared to FITC‐Chb‐M'‐exposed organs (Figure S3). In a sharp contrast, FITC signal from kidney was the strongest among FITC‐treated organs. These observations indicate that Chb‐M' selectively accumulates in cancers.

### 3.5 Chb‐M'‐Mediated Attenuation of Cancer Growth as Examined by CAM Assay

**FIGURE 5:**
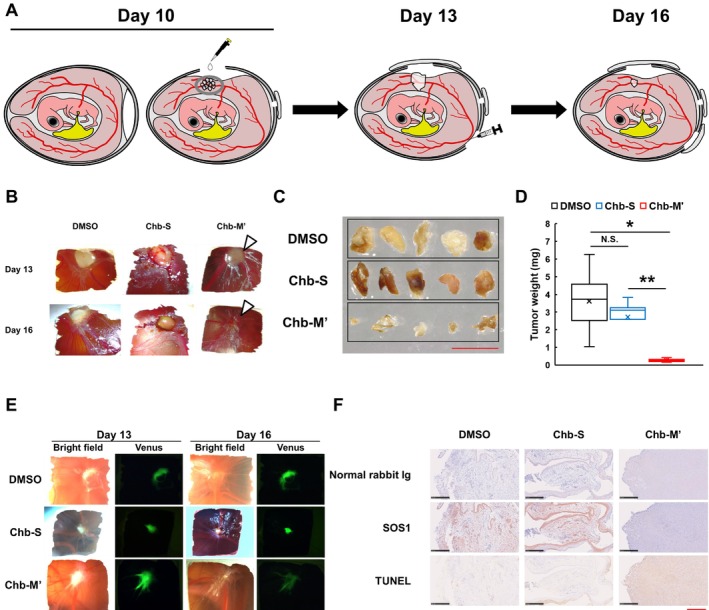
Chb‐M'‐mediated attenuation of cancer growth as examined by the CAM assay. (A) A graphical drawing of the chicken egg cancer model. On Day 13, the chicken eggs were intravenously injected with DMSO, Chb‐M', or with Chb‐S. On Day 16, the window of the eggs and the resected cancers developed on the CAM were observed with the stereomicroscope (*n* = 5). (B) Representative bright‐field images of the egg windows prepared from the indicated groups taken at Day 13 (upper) and 16 (lower). White arrowheads indicate nearly disappeared cancer cells in the Chb‐M' group. (C) Images of the resected CAM cancers prepared from each group (*n* = 5). Scale bars indicate 5 mm. (D) The weight of the CAM cancers resected from each experimental group (*n* = 5). The band, box, whiskers, and cross mark inside each box indicate the median, first, and third quartile points, minimum and maximum points, and mean, respectively. *p < 0.05, **p < 0.01; N.S., no significant (obtained by two‐tailed Student's *t*‐test). (E) Representative images showing Chb‐M'‐mediated growth inhibition of the CAM cancers arising from Venus‐expressing MKN45 cells. (F) Immunohistochemical staining. The resected CAM cancers prepared from each group at Day 16 were subjected to immunohistochemical staining with antihuman SOS1 antibody (middle panels) or with normal rabbit Ig (top panels). TUNEL staining was also performed (bottom panels). Scale bars indicate 250 μm.

As described previously, we have demonstrated that Chb‐M' has an in vivo anticancer effect on mouse xenograft model derived from MKN45 cells [10]. In this study, we asked whether in vivo anticancer effect of Chb‐M' could be reproduced in the CAM model. The experimental design was shown in Figure 5A. The eggshell was windowed and then MKN45 cells expressing Venus were transplanted onto the CAM (Day 10). On Day 13, Chb‐M' (6.4 μg/egg), Chb‐S (6.4 μg/egg), or DMSO (equivalent volume) was intravenously injected into the chicken eggs. On Day 16, the cancers developed on the CAM were observed under the stereomicroscope. As shown in Figure 5B, a remarkable cancer development was detectable on Day 13. Intriguingly, a significant inhibition of the CAM cancer growth was observed in Chb‐M'‐treated eggs but not in Chb‐S‐treated eggs (Day 16). In addition, the weight of the CAM cancer resected from Chb‐M'‐treated eggs was extremely smaller than that from Chb‐S‐exposed eggs (Figure 5C,D). In support of these results, Venus signal emitted from MKN45 cells was markedly reduced in Chb‐M'‐treated eggs but not in Chb‐S‐treated eggs (Figure 5E). In accordance with our previous results obtained from the mouse xenograft model [10, 17, 18, 19, 20, 21, 22, 23], our present results strongly suggest that Chb‐M'‐mediated attenuation of cancer growth is also detectable in the CAM model.

Since Chb‐M' was created to comprehensively suppress RUNX family, it is possible that RUNX family‐target gene expression could be decreased in response to Chb‐M'. According to our previous study [17], knockdown of *RUNX1* decreased the expression level of RTK adapter protein SOS1 (guanine nucleotide exchange factor) as well as the amount of phosphorylated ErbB2/HER2 in *HER2*‐amplified MKN45 and NUGC4 cells. Notably, we have also found that Chb‐M' suppresses the expression level of SOS1 and inhibits ErbB2/HER2 signaling pathway. As expected, our immunohistochemical analysis on the cancers developed on the CAM demonstrated that Chb‐M' but not Chb‐S downregulates the RUNX‐target gene product SOS1 (Figure 5F). Moreover, TUNEL‐positive apoptotic cells were readily detectable in the CAM cancers exposed to Chb‐M', whereas Chb‐S treatment had a negligible effect on the number of TUNEL‐positive cells in the CAM cancers.

Together, our findings collectively indicate that Chb‐M'‐induced cancer suppression is reproduced in the CAM model, which is mediated by downregulation of RUNX family‐target genes.

## 4 Discussion

In the present study, we have employed the chicken embryo‐based CAM system to evaluate the nuclear targeting, cancer accumulation, and anticancer abilities of PI polyamide termed Chb‐M' that targets the RUNX family consensus‐binding motif.

According to our present results, FITC‐Chb‐M' but not FITC‐Chb‐S was largely detectable within cell nucleus of human gastric cancer MKN45 and NUGC4 cells. Consistent with our recent observations obtained from mouse xenograft model [10], FITC‐Chb‐M' significantly accumulated in the CAM cancers, whereas FITC‐Chb‐M' was incorporated into various normal chicken organs but to a lesser extent.

From the clinical point of view, the specific accumulation of the candidate anticancer drugs such as Chb‐M' in cancers is quite important. It has been reported that most small molecules, such as Rhodamine B, have a tendency to distribute to all organs in the CAM model [7]. In contrast, the macromolecular drugs do not penetrate normal blood vessels and are likely to leak out through the imperfect peritumoral vessels, which is due to the enhanced permeation and retention (EPR) effects [27]. Since Chb‐M' is an intermediate molecule with a molecular weight of 1524.5, it is possible that the effect of EPR might be one of the causes of its cancer accumulation. Additionally, it is indicative that FITC‐Chb‐M' might escape from the blood circulation and then potentiate its cancer retention by its binding to DNA within the cancer cell nucleus through PI polyamide and chlorambucil.

Meanwhile, it has been shown that the extent of the nuclear distribution of PI polyamide is dependent on its chemical composition [28, 29]. Inoue et al. [29] described that the lower the number of imidazole within PI polyamide, the higher its hydrophobicity and the lower the renal excretion, the higher the cancer accumulation. Indeed, the number of imidazole within Chb‐M' and Chb‐S was 3 and 4, respectively. In addition, Chb‐M' was estimated to have approximately four times as many binding sites in the human genome as Chb‐S (Table S1). Thus, it is indicative that the decreased number of imidazole and increased number of DNA‐binding sites might contribute to the nuclear access of Chb‐M' relative to Chb‐S, and thus Chb‐M' displays a higher cancer retention than Chb‐S in the CAM model.

The CAM model significantly shortens the time required for cancer growth and reduces the costs (less than one dollar/egg) as compared to the conventional immunodeficient mouse model [1]. Thus, it is suggestive that the CAM assay system is suitable for the initial in vivo screening of the cancer accumulation and anticancer efficacy of various PI polyamides. More importantly, it has been shown that the clinical materials prepared from cancer patients, such as surgical specimens and biopsy samples, retain the ability to form CAM cancers that might mimic the patient's cancers [2, 3, 4, 5, 6]. Collectively, the CAM model might serve as a valuable and promising platform to rapidly establish the patient‐derived xenografts and evaluate the cancer uptake as well as anticancer efficacy of various PI polyamide‐based drug candidates [30].

## 5 Conclusion

By taking advantage of the CAM model, we have recapitulated the cancer accumulation and anticancer effect of our RUNX inhibitor Chb‐M'. Based on our present results, it is strongly suggested that the CAM model might serve as a valuable platform to rapidly assess cancer uptake and anticancer efficacy of various PI polyamide‐based drug candidates. Alternatively, it could also be possible to identify the metastasis foci by labeling the primary cancer with PI‐polyamide without anticancer effect. However, there are several differences between fertilized chicken eggs and mammals in terms of drug metabolism and immune systems. Therefore, it is suggestive that a combination of rapid screening in the CAM model followed by confirmation in mammals may be an accurate and effective approach.

## Author Contributions

**Tatsuya Masuda:** data curation (lead), formal analysis (lead), investigation (lead), methodology (lead), software (lead), validation (lead), visualization (lead), writing – original draft (equal). **Takayoshi Watanabe:** data curation (equal), formal analysis (equal), investigation (equal), methodology (equal), visualization (equal). **Yasutoshi Tatsumi:** data curation (supporting), writing – review and editing (equal). **Jason Lin:** investigation (equal), writing – review and editing (equal). **Kazuhiro Okumura:** investigation (equal). **Toshinori Ozaki:** writing – review and editing (equal). **Hiroshi Sugiyama:** resources (equal). **Yasuhiko Kamikubo:** conceptualization (lead), funding acquisition (lead), project administration (lead), resources (lead), supervision (lead), validation (lead).

## Conflicts of Interest

The authors declare no conflicts of interest.

## Supporting information


**Figure S1.** LC–MS assay of Chb‐S, Chb‐M’, FITC‐Chb‐S, and FITC‐Chb‐M’. The high‐performance liquid chromatography (LC‐2020, Shimadzu Industry) was employed to analyze the PI polyamides, using a 4.5 mm × 150 mm Phenomenex Gemini‐NX3u 5‐ODS‐H reverse‐phase column (Phenomenex) soaked in 0.1% acetic acid in water with acetonitrile as eluent, at a flow rate of 2 mL/min, and a linear gradient from 5% to 95% acetonitrile over 15 min, with detection at 310 nm. Chb‐S. *m/z* calculated for C_70_H_87_Cl_2_N_25_O_11_, [M + H]^+^ 1526.51; found 1526.05, [M + 2H]^2+^ 763.76; found 763.75. Chb‐M’. *m/z* calculated for C_71_H_88_Cl_2_N_24_O_11_, [M + H]^+^ 1525.52; found 1525.40, [M + 2H]^2+^ 763.26; found 763.25. FITC‐Chb‐S. *m/z* calculated for C_93_H_103_Cl_2_N_27_O_16_S, [M + H]^+^ 1958.96; found 1959.10, [M + 2H]^2+^ 979.98; found 979.95, [M + 3H]^3+^ 653.66; found 653.70. FITC‐Chb‐M’. *m/z* calculated for C_94_H_104_Cl_2_N_26_O_16_S, [M + H]^+^ 1957.97; found 1957.65, [M + 2H]^2+^ 979.49; found 979.10, [M + 3H]^3+^ 653.32; found 653.35.
**Figure S2.** Increased intra‐cancer uptake of FITC‐Chb‐M’ in fertilized chicken eggs. Bar graphs showed a time course of FITC fluorescence intensity arising from the CAM cancers exposed to FITC (A), FITC‐Chb‐S (B), or FITC‐Chb‐M’ (C) (*n* = 5). Data are the mean ± SEM values. A significant difference test was performed between the signal intensities at 3 and 24 h after injection. *p < 0.05; N.S., not significant (two‐tailed Student’s *t*‐test).
**Figure S3.** Preferential cancer distribution of FITC‐Chb‐M’. Representative bright‐field and FITC fluorescence (taken with GFP filter) images. The chicken eggs were treated as in Figure 3A. Twenty‐four hours after injection, the embryonic organs and cancers prepared from the indicated groups were observed under the fluorescence stereomicroscope. The arrangement of cancers and each organ are shown (lower right). Red or white arrowhead indicates the signal arising from FITC‐Chb‐M’‐ or other drug‐treated CAM cancers, respectively.

**Table S1.** Binding sequences of Chb‐M’ and Chb‐S, and the number of their respective binding sites in the human genome.

## Data Availability

The data that support the findings of this study are available from the corresponding author (YK) upon reasonable request.

## References

[1] A. Komatsu , Y. Higashi , and K. Matsumoto , “Various CAM Tumor Models,” Enzymes 46 (2019): 37–57, 10.1016/bs.enz.2019.10.001. 31727276

[2] A. Komatsu , K. Matsumoto , T. Saito , M. Muto , and F. Tamanoi , “Patient Derived Chicken Egg Tumor Model (PDcE Model): Current Status and Critical Issues,” Cells 8, no. 5 (2019): 440. 31083409 10.3390/cells8050440 PMC6562823

[3] G. Sys , M. Van Bockstal , R. Forsyth , et al., “Tumor Grafts Derived From Sarcoma Patients Retain Tumor Morphology, Viability, and Invasion Potential and Indicate Disease Outcomes in the Chick Chorioallantoic Membrane Model,” Cancer Letters 326, no. 1 (2012): 69–78. 22841668 10.1016/j.canlet.2012.07.023

[4] L. C. DeBord , R. R. Pathak , M. Villaneuva , et al., “The Chick Chorioallantoic Membrane (CAM) as a Versatile Patient‐Derived Xenograft (PDX) Platform for Precision Medicine and Preclinical Research,” American Journal of Cancer Research 8, no. 8 (2018): 1642–1660. 30210932 PMC6129484

[5] J. Hu , M. Ishihara , A. I. Chin , and L. Wu , “Establishment of Xenografts of Urological Cancers on Chicken Chorioallantoic Membrane (CAM) to Study Metastasis,” Precision Clinical Medicine 2, no. 3 (2019): 140–151. 31598385 10.1093/pcmedi/pbz018 PMC6770283

[6] A. Komatsu , K. Matsumoto , Y. Yoshimatsu , et al., “The CAM Model for *CIC‐DUX4* Sarcoma and Its Potential Use for Precision Medicine,” Cells 10, no. 10 (2021): 2613. 34685592 10.3390/cells10102613 PMC8533847

[7] Y. Higashi , S. Ikeda , K. Matsumoto , et al., “Tumor Accumulation of PIP‐Based KRAS Inhibitor KR12 Evaluated by the Use of a Simple, Versatile Chicken Egg Tumor Model,” Cancers (Basel) 14, no. 4 (2022): 951. 35205697 10.3390/cancers14040951 PMC8869854

[8] T. Okuda , J. van Deursen , S. W. Hiebert , G. Grosveld , and J. R. Downing , “AML1, the Target of Multiple Chromosomal Translocations in Human Leukemia, Is Essential for Normal Fetal Liver Hematopoiesis,” Cell 84, no. 2 (1996): 321–330. 8565077 10.1016/s0092-8674(00)80986-1

[9] R. Sood , Y. Kamikubo , and P. Liu , “Role of RUNX1 in Hematological Malignancies,” [Published Correction Appears in *Blood* 131, no. 3 (January 18, 2018): 373] Blood 129, no. 15 (2017): 2070–2082. 28179279 10.1182/blood-2016-10-687830 PMC5391618

[10] K. Morita , K. Suzuki , S. Maeda , et al., “Genetic Regulation of the RUNX Transcription Factor Family Has Antitumor Effects,” Journal of Clinical Investigation 127, no. 7 (2017): 2815–2828. 28530640 10.1172/JCI91788 PMC5490777

[11] Y. Kamikubo , “Genetic Compensation of RUNX Family Transcription Factors in Leukemia,” Cancer Science 109, no. 8 (2018): 2358–2363, 10.1111/cas.13664. 29883054 PMC6113440

[12] Y. Kamikubo , “CROX (Cluster Regulation of RUNX) as a Potential Novel Therapeutic Approach,” Molecules and Cells 43, no. 2 (2020): 198–202. 31991534 10.14348/molcells.2019.0268 PMC7057841

[13] R. Maeda , S. Sato , S. Obata , et al., “Molecular Characteristics of DNA‐Alkylating PI Polyamides Targeting RUNX Transcription Factors,” Journal of the American Chemical Society 141, no. 10 (2019): 4257–4263. 30601664 10.1021/jacs.8b08813

[14] B. H. Ganesh , B. Aruchamy , P. Ramani , et al., “Exploration of N‐Tetra‐Substituted Imidazoles as Effective Agents to Counteract Gastric Cancer Cell Viability: Synthesis and Biological Evaluation,” ChemistrySelect 8 (2023): e202302768.

[15] L. Chen , N. Fukuda , T. Ueno , M. Abe , and T. Matsumoto , “Development of Multifunctional Pyrrole‐Imidazole Polyamides That Increase Hepatocyte Growth Factor and Suppress Transforming Growth Factor‐β1,” Journal of Pharmacological Sciences 154, no. 1 (2024): 1–8, 10.1016/j.jphs.2023.11.001. 38081679

[16] B. H. Ganesh , A. G. Raj , B. Aruchamy , P. Nanjan , C. Drago , and P. Ramani , “Pyrrole: A Decisive Scaffold for the Development of Therapeutic Agents and Structure‐Activity Relationship,” ChemMedChem 19 (2024): e202300447. 37926686 10.1002/cmdc.202300447

[17] Y. Mitsuda , K. Morita , G. Kashiwazaki , et al., “RUNX1 Positively Regulates the ErbB2/HER2 Signaling Pathway Through Modulating SOS1 Expression in Gastric Cancer Cells,” Scientific Reports 8, no. 1 (2018): 6423. 29686309 10.1038/s41598-018-24969-w PMC5913281

[18] T. Masuda , S. Maeda , S. Shimada , et al., “RUNX1 Transactivates BCR‐ABL1 Expression in Philadelphia Chromosome Positive Acute Lymphoblastic Leukemia,” Cancer Science 113, no. 2 (2022): 529–539. 34902205 10.1111/cas.15239 PMC8819354

[19] T. Daifu , M. Mikami , H. Hiramatsu , et al., “Suppression of Malignant Rhabdoid Tumors Through Chb‐M'‐Mediated RUNX1 Inhibition,” Pediatric Blood & Cancer 68, no. 2 (2021): e28789. 33180377 10.1002/pbc.28789

[20] M. Mikami , T. Masuda , T. Kanatani , et al., “RUNX1‐Survivin Axis Is a Novel Therapeutic Target for Malignant Rhabdoid Tumors,” Molecules and Cells 45, no. 12 (2022): 886–895. 36572559 10.14348/molcells.2022.2031 PMC9794559

[21] T. Masuda , T. Daifu , M. Mikami , et al., “Cluster Regulation of RUNX (CROX): Strategy Against Malignant Rhabdoid Tumor,” Journal of Blood & Lymph 13, no. 1 (2023), 10.37421/2165-7831.2023.13.296.

[22] E. Y. Hattori , T. Masuda , Y. Mineharu , et al., “A RUNX‐Targeted Gene Switch‐Off Approach Modulates the BIRC5/PIF1‐p21 Pathway and Reduces Glioblastoma Growth in Mice,” [Published Correction Appears in *Communications Biology* 5, no. 1 (September 27, 2022): 1021] Communications Biology 5, no. 1 (2022): 939. 36085167 10.1038/s42003-022-03917-5 PMC9463152

[23] Y. Matsui , Y. Mineharu , Y. Noguchi , et al., “Chlorambucil‐Conjugated PI‐Polyamides (Chb‐M'), a Transcription Inhibitor of RUNX Family, Has an Anti‐Tumor Activity Against SHH‐Type Medulloblastoma With p53 Mutation,” [Published Correction Appears in *Biochemical and Biophysical Research Communications* (March 23, 2023)] Biochemical and Biophysical Research Communications 620 (2022): 150–157. 35792512 10.1016/j.bbrc.2022.06.090

[24] H. Kubota , T. Masuda , M. Noura , et al., “RUNX Inhibitor Suppresses Graft‐Versus‐Host Disease Through Targeting *RUNX‐NFATC2* Axis,” EJHaem 2, no. 3 (2021): 449–458. 35844683 10.1002/jha2.230 PMC9175814

[25] Y. Hirose , S. Sato , K. Hashiya , T. Bando , and H. Sugiyama , “Anticancer Activities of DNA‐Alkylating Pyrrole–Imidazole Polyamide Analogs Targeting RUNX Transcription Factors Against p53‐Mutated Pancreatic Cancer PANC‐1 Cells,” Journal of Medicinal Chemistry 66, no. 17 (2023): 12059–12068, 10.1021/acs.jmedchem.3c00613. 37606185

[26] S. Asamitsu , Y. Kawamoto , F. Hashiya , et al., “Sequence‐Specific DNA Alkylation and Transcriptional Inhibition by Long‐Chain Hairpin Pyrrole‐Imidazole Polyamide‐Chlorambucil Conjugates Targeting CAG/CTG Trinucleotide Repeats,” Bioorganic & Medicinal Chemistry 22, no. 17 (2014): 4646–4657. 25127467 10.1016/j.bmc.2014.07.019

[27] P. Gawali , A. Saraswat , S. Bhide , S. Gupta , and K. Patel , “Human Solid Tumors and Clinical Relevance of the Enhanced Permeation and Retention Effect: A ‘Golden Gate’ for Nanomedicine in Preclinical Studies?,” Nanomedicine 18, no. 2 (2023): 169–190. 37042320 10.2217/nnm-2022-0257

[28] T. P. Best , B. S. Edelson , N. G. Nickols , and P. B. Dervan , “Nuclear Localization of Pyrrole‐Imidazole Polyamide‐Fluorescein Conjugates in Cell Culture,” Proceedings of the National Academy of Sciences of the United States of America 100, no. 21 (2003): 12063–12068. 14519850 10.1073/pnas.2035074100 PMC218713

[29] T. Inoue , O. Shimozato , N. Matsuo , et al., “Hydrophobic Structure of Hairpin Ten‐Ring Pyrrole‐Imidazole Polyamides Enhances Tumor Tissue Accumulation/Retention In Vivo,” Bioorganic & Medicinal Chemistry 26, no. 9 (2018): 2337–2344. 29622411 10.1016/j.bmc.2018.03.029

[30] D. Ribatti , “The Chick Embryo Chorioallantoic Membrane Patient‐Derived Xenograft (PDX) Model,” Pathology, Research and Practice 243 (2023): 154367. 36774760 10.1016/j.prp.2023.154367

