# Supplementary material for: Cancer Accumulation and Anticancer Activity of “CROX (Cluster Regulation of RUNX)” PIP in HER2 ‐Positive Gastric Cancer Evaluated by Chicken Egg Cancer Model
Source: Cancer Med. 2025 Apr 2;14(7):e70845. doi: 10.1002/cam4.70845 (PMC11962651; doi:10.1002/cam4.70845)
Supplement: Supplementary file 1 — Figure S1. LC–MS assay of Chb‐S, Chb‐M’, FITC‐Chb‐S, and FITC‐Chb‐M’. The high‐performance liquid chromatography (LC‐2020, Shimadzu Industry) was employed to analyze the PI polyamides, using a 4.5 mm × 150 mm Phenomenex Gemini‐NX3u 5‐ODS‐H reverse‐phase column (Phenomenex) soaked in 0.1% acetic acid in water with acetonitrile as eluent, at a flow rate of 2 mL/min, and a linear gradient from 5% to 95% acetonitrile over 15 min, with detection at 310 nm. Chb‐S. m/z calculated for C70H87Cl2N25O11, [M + H]+ 1526.51; found 1526.05, [M + 2H]2+ 763.76; found 763.75. Chb‐M’. m/z calculated for C71H88Cl2N24O11, [M + H]+ 1525.52; found 1525.40, [M + 2H]2+ 763.26; found 763.25. FITC‐Chb‐S. m/z calculated for C93H103Cl2N27O16S, [M + H]+ 1958.96; found 1959.10, [M + 2H]2+ 979.98; found 979.95, [M + 3H]3+ 653.66; found 653.70. FITC‐Chb‐M’. m/z calculated for C94H104Cl2N26O16S, [M + H]+ 1957.97; found 1957.65, [M + 2H]2+ 979.49; found 979.10, [M + 3H]3+ 653.32; found 653.35. Figure S2. Increased intra‐cancer uptake of FITC‐Chb‐M’ in fertilized chicken eggs. Bar graphs showed a time course of FITC fluorescence intensity arising from the CAM cancers exposed to FITC (A), FITC‐Chb‐S (B), or FITC‐Chb‐M’ (C) (n = 5). Data are the mean ± SEM values. A significant difference test was performed between the signal intensities at 3 and 24 h after injection. *p < 0.05; N.S., not significant (two‐tailed Student’s t‐test). Figure S3. Preferential cancer distribution of FITC‐Chb‐M’. Representative bright‐field and FITC fluorescence (taken with GFP filter) images. The chicken eggs were treated as in Figure 3A. Twenty‐four hours after injection, the embryonic organs and cancers prepared from the indicated groups were observed under the fluorescence stereomicroscope. The arrangement of cancers and each organ are shown (lower right). Red or white arrowhead indicates the signal arising from FITC‐Chb‐M’‐ or other drug‐treated CAM cancers, respectively. [file CAM4-14-e70845-s002.pdf]

Figure S1.

Chb-S

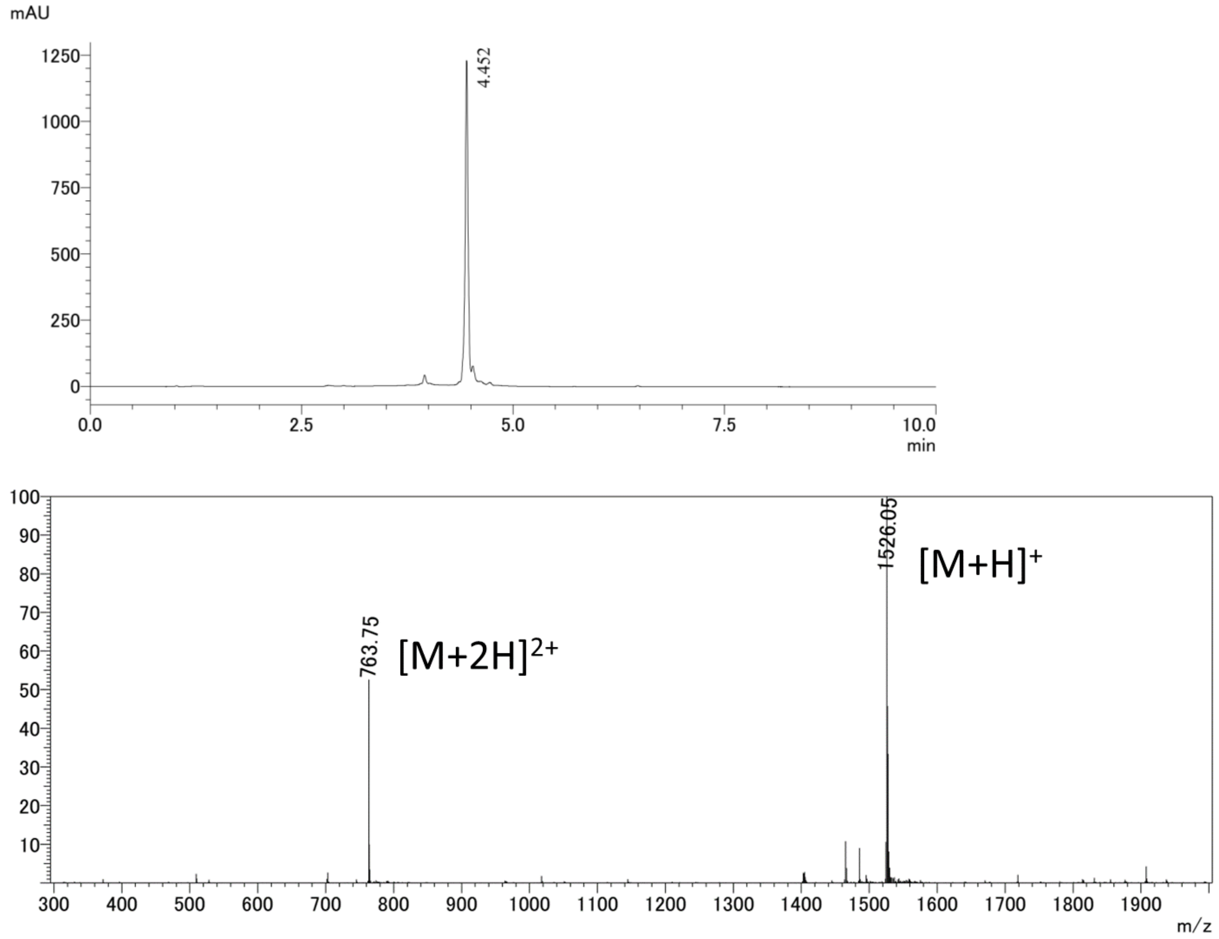

Chb-M'

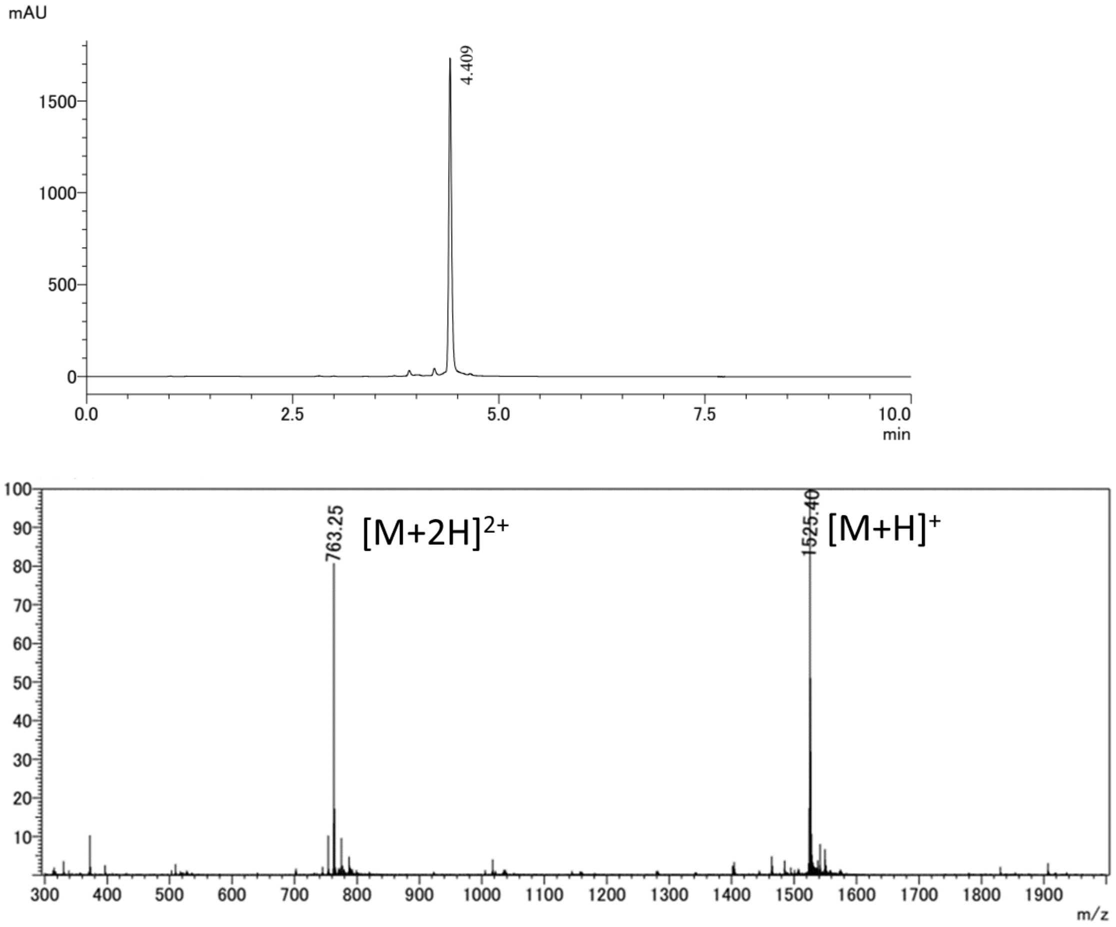

FITC-Chb-S

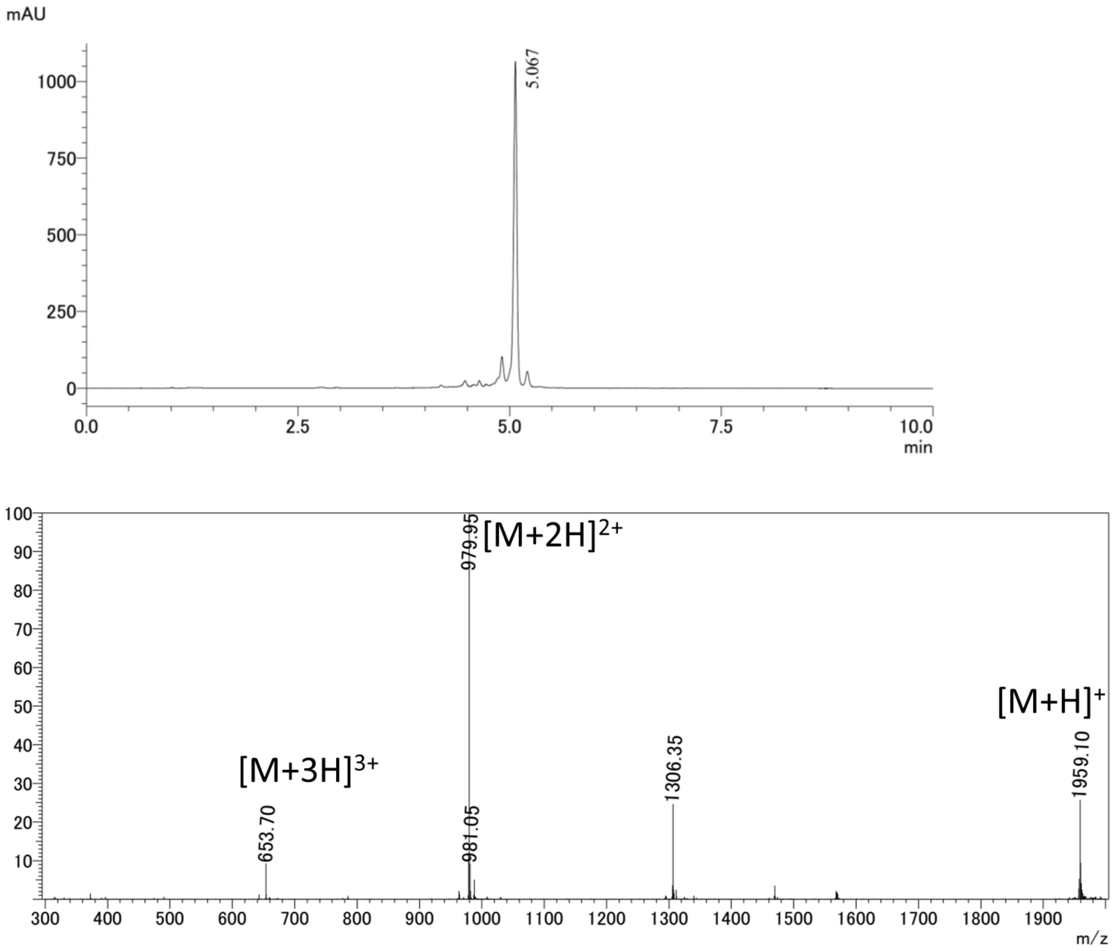

FITC-Chb-M'

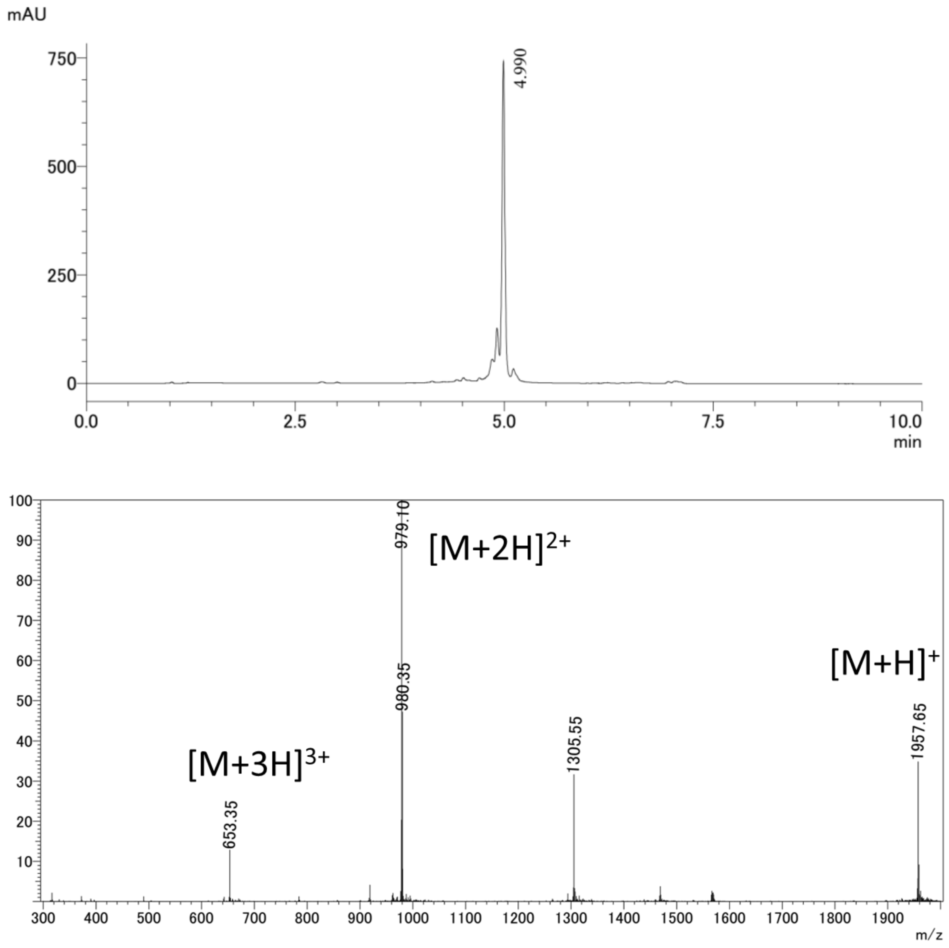

Figure S2.

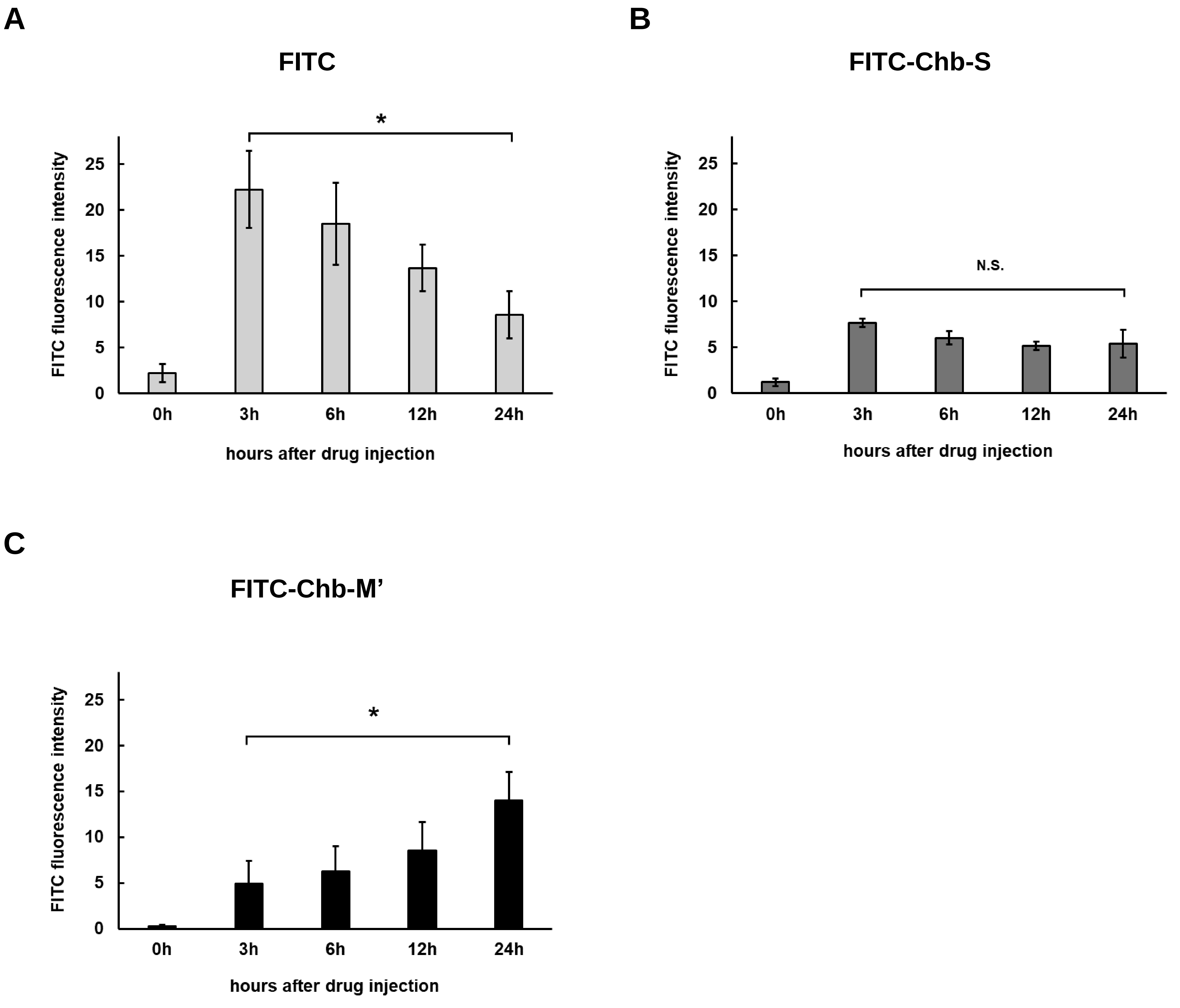
A
FITC
*
FITC fluorescence intensity
0
5
10
15
20
25
0h
3h
6h
12h
24h
hours after drug injection
B
FITC-Chb-S
N.S.
FITC fluorescence intensity
0
5
10
15
20
25
0h
3h
6h
12h
24h
hours after drug injection
C
FITC-Chb-M’
*
FITC fluorescence intensity
0
5
10
15
20
25
0h
3h
6h
12h
24h
hours after drug injection

Figure S3.

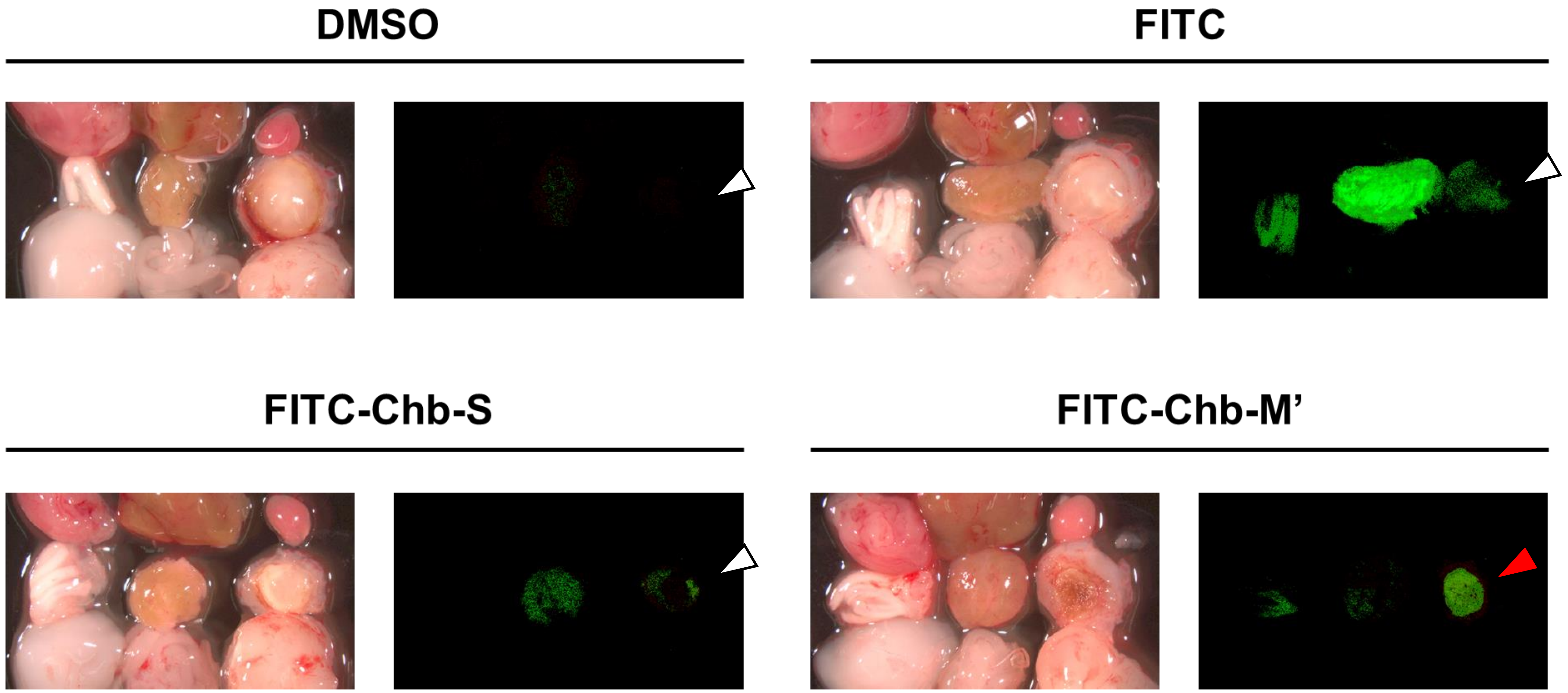

Heart	Liver	Spleen
Lung	Kidney	Tumor
Brain	Intestine	Stomach
