# Supplementary material for: Cancer Accumulation and Anticancer Activity of “CROX (Cluster Regulation of RUNX)” PIP in HER2 ‐Positive Gastric Cancer Evaluated by Chicken Egg Cancer Model
Source: Cancer Med. 2025 Apr 2;14(7):e70845. doi: 10.1002/cam4.70845 (PMC11962651; doi:10.1002/cam4.70845)
Supplement: Supplementary file 2 — Table S1. Binding sequences of Chb‐M’ and Chb‐S, and the number of their respective binding sites in the human genome. [file CAM4-14-e70845-s001.pptx]

## Slide 1
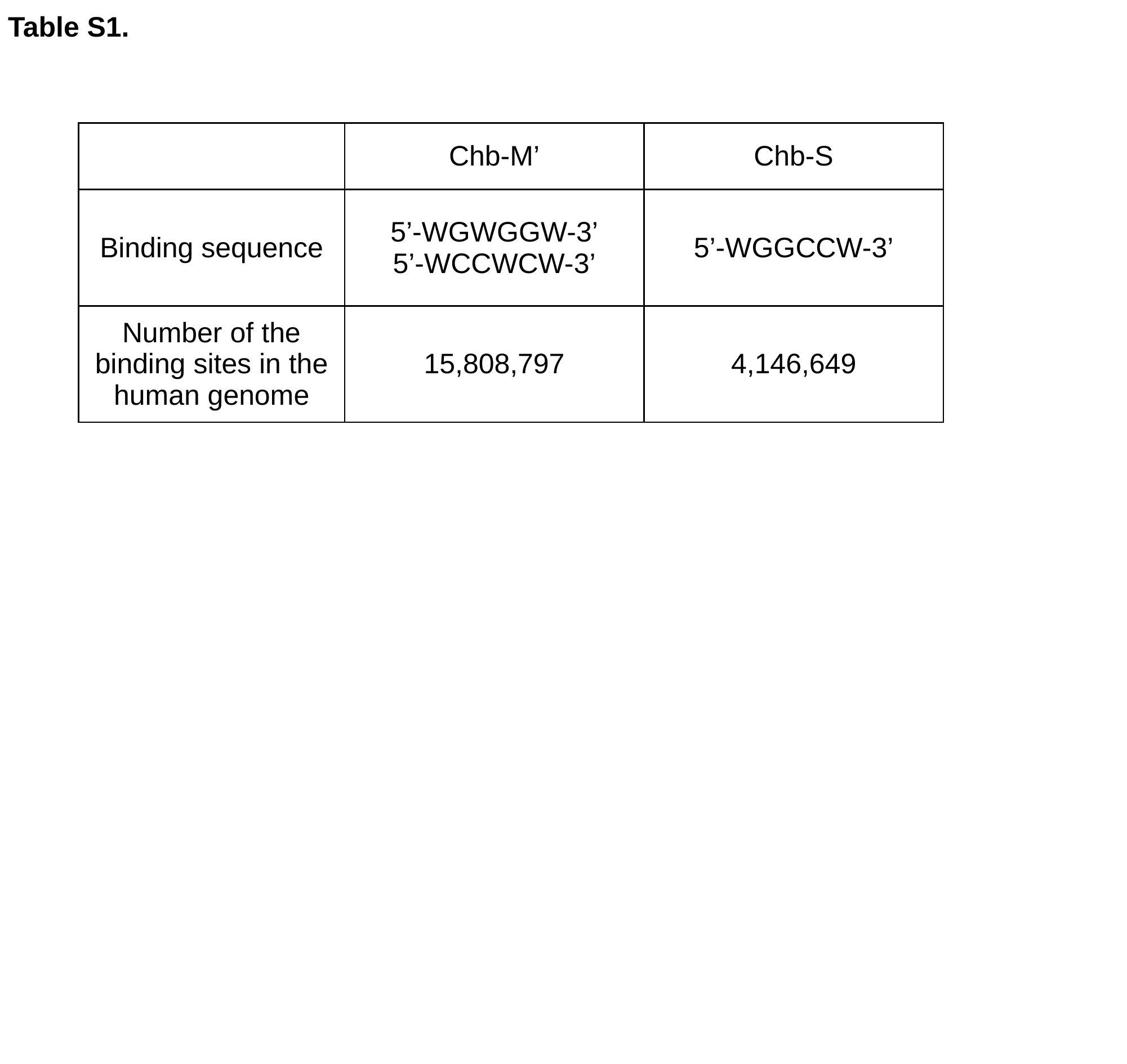

Table S1.
| | Chb-M’ | Chb-S |
| --- | --- | --- |
| Binding sequence | 5’-WGWGGW-3’ 5’-WCCWCW-3’ | 5’-WGGCCW-3’ |
| Number of the binding sites in the human genome | 15,808,797 | 4,146,649 |
